# Unveiling Giant Onychomatricoma: A Diagnostic and Therapeutic Challenge

**DOI:** 10.1155/crdm/7190051

**Published:** 2026-02-05

**Authors:** Katherine Nicole Calderón Tiburcio, Winston Damian Brito, Mariel Isa Pimentel, Ana Arisleyda Núñez

**Affiliations:** ^1^ Department of Dermatology, Instituto Dermatologico y Cirugia de Piel Dr. Huberto Bogaert Diaz, Santo Domingo, Dominican Republic

**Keywords:** nail disorders, nail tumors, onychomatrichoma, onychomycosis

## Abstract

Onychomatricoma is a rare benign tumor of the nail matrix that is often misdiagnosed due to its resemblance to other nail conditions, particularly onychomycosis. A giant variant, which affects the entire nail structure, is even more uncommon, with fewer than 20 cases documented. We present the case of a 62‐year‐old male with a 5‐year history of a slow‐growing, asymptomatic tumor on the third nail of his left hand. Clinical examination revealed a papillomatous mass originating from the nail matrix, accompanied by nail thickening and xanthonychia. Dermoscopy showed longitudinal yellow, white, and gray lines, splinter hemorrhages, and a honeycomb pattern. Mycological testing suggested onychomycosis, but due to the size and persistence of the lesion, a multidisciplinary team recommended a complete nail avulsion and proximal matricectomy. Histopathological analysis revealed fibroepithelial projections covered by squamous epithelium and thickened nail plate fragments, confirming the diagnosis of giant onychomatricoma. The patient underwent surgical management and has shown no signs of recurrence after 2 years of follow‐up. This case highlights the diagnostic and therapeutic challenges posed by giant onychomatricoma, particularly when coexisting with fungal infection. It also underscores the need for thorough clinical, dermoscopic, and histopathological evaluation in cases of chronic nail deformities.

## 1. Introduction

Onychomatricoma is a rare benign tumor that grows from the nail matrix. While its cause remains unknown, trauma and fungal nail infections are frequently associated [1, 2]. Baran and Kint first described this tumor in 1992, and fewer than 200 cases have been reported since then [3]. In 2007, Estrada‐Chávez and colleagues [4] identified an even rarer giant form that affects the entire nail structure. To date, fewer than 20 giant cases have been documented [3]. Here, we present a diagnostically challenging case of giant onychomatricoma in a male patient with a 5‐year asymptomatic evolution, initially mistaken for onychomycosis. The coexistence of fungal infection and a slow‐growing tumor highlights the diagnostic pitfalls clinicians may encounter, especially in resource‐limited settings where advanced imaging or biopsy may not be immediately available [4]. This case underscores the importance of maintaining a high index of suspicion in persistent nail abnormalities and contributes to the limited clinical evidence surrounding this rare entity.

## 2. Clinical Case

A 62‐year‐old male with a known history of hypertension, presented to our clinic with a 5‐year history of a tumor on his left hand’s third nail. He could not recall any previous local trauma and denied any symptoms. Clinical examination revealed a 1.5 × 2 cm papillomatous mass originating from the nail matrix, with marked nail plate thickening and deformation and xanthonychia (Figure 1(a)). The dermoscopy revealed longitudinal yellow, white, and gray lines, splinter hemorrhages, and black spots that formed a honeycomb pattern in the nail plate (Figure 1(b)). Direct mycological investigation indicated the presence of filaments consistent with onychomycosis. A clinical diagnosis of onychomycosis rather than onychomatricoma was obtained. A multidisciplinary team consisting of two mycologists and a surgeon evaluated the patient and determined that the best approach was a diagnostic and therapeutic complete avulsion of the nail plate along with proximal matricectomy. Although matricectomy is not routinely considered first‐line treatment, the team recommended it due to the tumor’s size, its cosmetic impact, and the patient’s occupation, which required frequent hand use. The patient also expressed a preference for a definitive solution to minimize the risk of recurrence.

FIGURE 1(a) Papillomatous mass at the nail matrix with nail thickening, internal projections, and xanthonychia. (b) Typical dermoscopic honeycomb pattern in onychomatrichoma revealing longitudinal yellow, white, and gray lines, splinter hemorrhages, and black spots.

**Figure:**
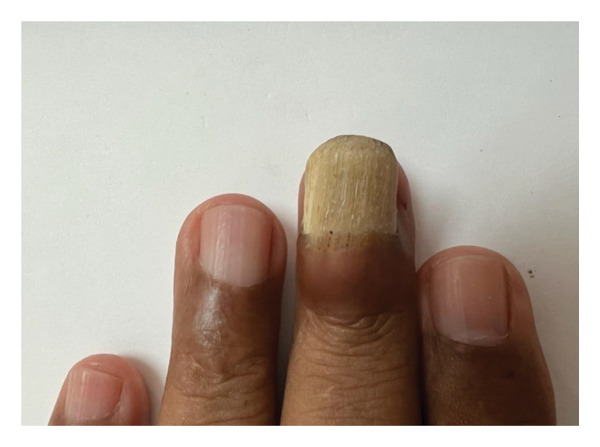
(a)

**Figure:**
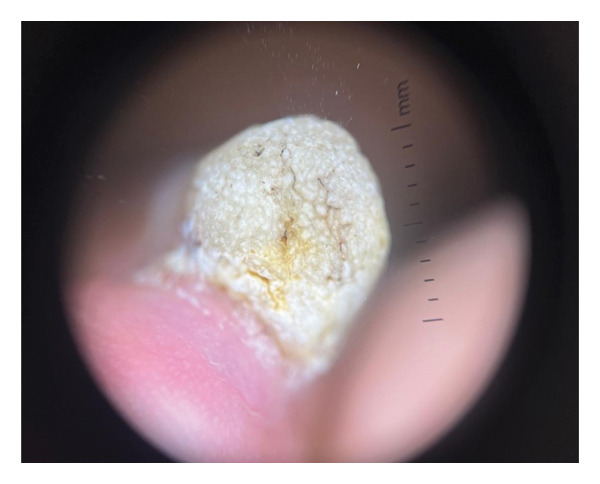
(b)

**FIGURE 3:**
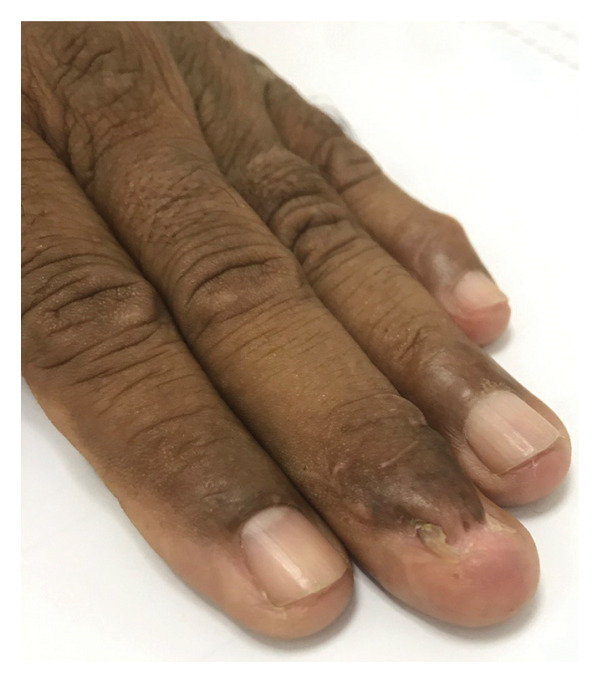
Clinical photograph at 7‐month follow‐up showing complete regrowth of the nail plate with no evidence of recurrence or dystrophy.

Further histopathological analysis revealed numerous fibroepithelial projections covered by squamous epithelium lacking a granular layer, as well as nail plate thickening, findings consistent with onychomatricoma (Figure 2(a)). Fragments from the nail plate and a significant thickening of the nail plate were also observed (Figure 2(b)). The patient underwent complete surgical excision of the lesion, including nail plate avulsion and proximal matricectomy under digital nerve block. Postoperative care included daily antiseptic dressing and oral antibiotics for 7 days. The healing process was uneventful, and the nail bed re‐epithelialized within 4 weeks. At the 7‐month follow‐up, there was no evidence of recurrence and, except for a small spicule, no nail regrowth. Photographic follow‐up images show full resolution of the lesion (Figure 3).

FIGURE 2(a) Hematoxylin and eosin (HE) 40x: fibroepithelial projections and thickened nail plate fragments covered by squamous epithelium lacking a granular layer. (b) HE 40x: fragments from the nail plate and nail plate thickening.

**Figure:**
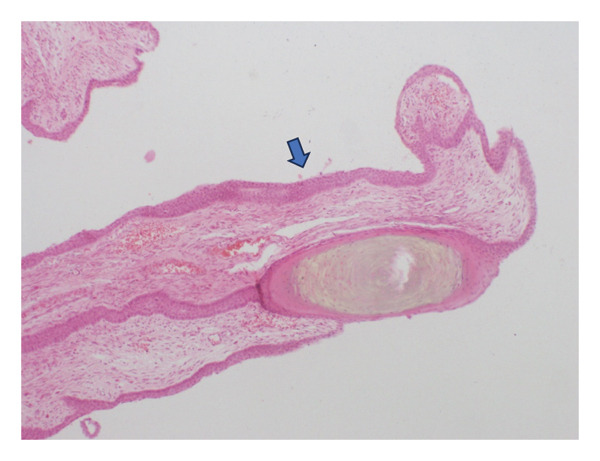
(a)

**Figure:**
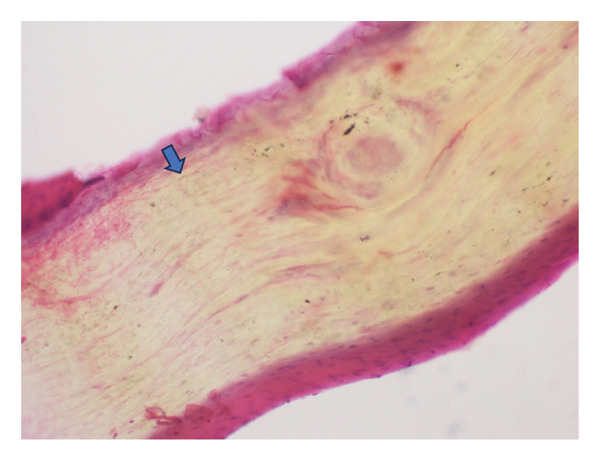
(b)

## 3. Discussion

Onychomatricoma is a rare filamentous tumor of the matrix affecting the nail [2]. It tends to be slow‐growing, asymptomatic, and often misdiagnosed as onychomycosis. Typically, it presents in the fifth or sixth decade of life and is more commonly seen in women [2]. It is classified as a benign ungual tumor and, along with the onychopapilloma, onychocytic carcinoma, and onychocytic matricoma, integrates the nail matrix tumors subgroup [5]. Its origin is controversial but previous trauma and onychomycosis have been associated; however, its causality role remains to be determined [1]. These tumors can produce keratin autonomously in the nail plate, which can contribute to both deformation and secondary fungi invasion. Giant onychomatricoma is a rarer and more exuberant variant; although there is no consensus on the dimensions to this variant, most published cases involve the entire nail unit or cause noticeable deformity [6]. The diagnosis is made on clinical and histopathological examinations; at inspection, the affected nail usually presents xanthonychia in the longitudinal segment of the nail, paronychia, increased longitudinal and transverse curvature and splinter hemorrhages. Melanonychia, erythronychia, dorsal pterygium, and fracture of the nail plate have been described [1, 7]. Histologically, onychomatricoma is a fibroepithelial tumor characterized by papillomatous projections lined by matrix epithelium and supported by a fibrous stroma. While some authors describe proximal and distal zones based on anatomic position and growth pattern, this division is not always well demarcated. The proximal portion, often located beneath the posterior nail fold, shows epithelial invaginations and a V‐shaped hyperkeratogenous zone. Distally, epithelial digitations extend outward, creating cavities within the overlying nail plate that may resemble penetration but actually result from the papillomatous projections covered by epithelium. These changes contribute to the characteristic “Swiss cheese” appearance of the nail plate observed in some cases [1]. While the latter is a classic feature of onychomatricoma, they were not evident in our case. This variation may reflect differences in tumor architecture or degree of nail plate involvement and underscores the spectrum of presentations associated with this entity. Differential diagnosis includes a wide variety of tumors: subungual keratoacanthoma, amelanotic melanoma, subungual squamous cell carcinoma, fibrokeratoma, subungual porocarcinoma, exostoses, and warts. Its association with onychomycosis is reported in the literature; whether onychomatricoma is a risk factor to develop onychomycosis or is a consequence of the fungal infection is unknown but both hypotheses have been proposed [1]. Dermoscopy can be helpful for the diagnosis; the most frequent findings are subungual nail thickening, splinter hemorrhages, honeycomb‐like cavities, and parallel white lines [1, 5]. Imaging techniques such as ultrasonography, magnetic resonance imaging (MRI), radiography, to rule out bone involvement or malignancy, and confocal microscopy have been described as useful tools in the diagnosis of onychomatricoma, especially for assessing tumor depth and nail matrix involvement [8]. Ultrasound, in particular, can reveal hypoechoic tumor projections and matrix thickening with high resolution [9]. However, in our case, these modalities were not employed due to limited access, economic constraints, and the lack of trained specialists with experience in nail unit ultrasonography. The diagnosis was, therefore, made clinically and confirmed through histopathological examination following surgical excision. The gold standard for management is complete surgical excision, especially when the nail has concomitant nail infection [1, 6]. This should include the proximal matrix to prevent local recurrence. Our case shares clinical and dermoscopic characteristics with previously reported onychomatricoma cases, such as yellowish discoloration, splinter hemorrhages, and papillomatous projections [1]. However, it presents several distinctive features. Compared with the original series described by Baran and Kint, which involved typical, smaller tumors, our case corresponds to the giant variant, a form scarcely reported in the literature [4, 5]. Most giant onychomatricomas have been documented in women, whereas our patient was male, aligning only partially with cases such as Gomes et al. [1] Additionally, our patient’s 5‐year asymptomatic evolution is among the longest reported, and the presence of concurrent onychomycosis complicated the clinical picture, initially leading to misdiagnosis and delay in definitive treatment. Although rare, this overlap with fungal infection has been reported and reflects the diagnostic pitfalls previously noted in similar cases [10]. The therapeutic outcome, however, was favorable, with nail regrowth and no recurrence at 7 months postsurgery.

## Funding

No funding was received for this manuscript.

## Conflicts of Interest

The authors declare no conflicts of interest.

## Data Availability

All data generated or analyzed during this study are included within this published article. Further inquiries can be directed to the corresponding author.
